# Generation of Marker-Free *pbd-2* Knock-in Pigs Using the CRISPR/Cas9 and Cre/loxP Systems

**DOI:** 10.3390/genes11080951

**Published:** 2020-08-18

**Authors:** Jing Huang, Antian Wang, Chao Huang, Yufan Sun, Bingxiao Song, Rui Zhou, Lu Li

**Affiliations:** 1 State Key Laboratory of Agricultural Microbiology, College of Veterinary Medicine, Huazhong Agricultural University, Wuhan 430070, China; isaac286@live.com (J.H.); hc394511335@163.com (C.H.); 2 Cooperative Innovation Center for Sustainable Pig Production, Huazhong Agricultural University, Wuhan 430070, China; wangantian@webmail.hzau.edu.cn (A.W.); leoandff@webmail.hzau.edu.cn (Y.S.); songbingxiao@webmail.hzau.edu.cn (B.S.); 3 Key Laboratory of Development of Veterinary Diagnostic Products, Ministry of Agriculture and Rural Affairs of China, Wuhan 430070, China; 4 International Research Center for Animal Disease, Ministry of Science and Technology of China, Wuhan 430070, China

**Keywords:** porcine β-defensin 2, CRISPR/Cas9, transgenic pigs, antimicrobial peptide, disease-resistant animals

## Abstract

Porcine β-defensin 2 (PBD-2), expressed by different tissues of pigs, is a multifunctional cationic peptide with antimicrobial, immunomodulatory and growth-promoting abilities. As the latest generation of genome-editing tool, CRISPR/Cas9 system makes it possible to enhance the expression of PBD-2 in pigs by site-specific knock-in of *pbd-2* gene into the pig genome. In this study, we aimed to generate marker-free *pbd-2* knock-in pigs using the CRISPR/Cas9 and Cre/loxP systems. Two copies of *pbd-2* gene linked by a T2A sequence were inserted into the porcine *Rosa26* locus through CRISPR/Cas9-mediated homology-directed repair. The floxed selectable marker gene *neoR*, used for G418 screening of positive cell clones, was removed by cell-penetrating Cre recombinase with a recombination efficiency of 48.3%. Cloned piglets were produced via somatic cell nuclear transfer and correct insertion of *pbd-2* genes was confirmed by PCR and Southern blot. Immunohistochemistry and immunofluorescence analyses indicated that expression levels of PBD-2 in different tissues of transgenic (TG) piglets were significantly higher than those of their wild-type (WT) littermates. Bactericidal assays demonstrated that there was a significant increase in the antimicrobial properties of the cell culture supernatants of porcine ear fibroblasts from the TG pigs in comparison to those from the WT pigs. Altogether, our study improved the protein expression level of PBD-2 in pigs by site-specific integration of *pbd-2* into the pig genome, which not only provided an effective pig model to study the anti-infection mechanisms of PBD-2 but also a promising genetic material for the breeding of disease-resistant pigs.

## 1. Introduction

Different breeding techniques have been utilized to improve animal production traits including growth rate, milk yield and disease-resistance. With the rapid development of biotechnologies, transgenic (TG) techniques have been gradually applied in animal breeding. Notably, there are three major gene-editing tools used, named as zinc finger nuclease, transcription activator-like effector nuclease and CRISPR/Cas9 [1,2,3]. Events of using gene-editing tools to improve quantitative traits and welfare of animals, and to eliminate allergens in livestock products, have been well described. Previous studies showed that knock-out of myostatin gene in cattle, goats, pigs and rabbits greatly increased their muscle mass [4,5,6]. Hornless cattle were produced through integrating *POLLED* gene into the genome, which prevented the suffering of cattle caused by dehorning [7]. Eggs with low allergenicity were produced by gene disruptions of *OVA* and *OVM* genes in hens [8], while β-lactoglobulin-free goat milk was obtained by removing *BLG* gene in goats [9].

The gene-editing technologies have also been widely used to improve disease-resistance in pigs [10]. For instance, pigs expressing porcine reproductive and respiratory syndrome virus (PRRSV)-specific small interfering RNA (siRNA) displayed enhanced resistance to PRRSV infection [11]. Similarly, pigs resistant to foot-and-mouth disease and classical swine fever (CSF) were obtained by inserting a corresponding siRNA expression cassette into the pig genome, respectively [12,13]. The deletion of CD163 SRCR5 domain conferred resilience to PRRSV in pigs [14], while pigs lacking aminopeptidase N acquired insusceptibility to transmissible gastroenteritis virus infection [15]. In addition, it has been identified that overexpression of porcine β-defensin 2 (PBD-2) and histone deacetylase 6 can protect pigs from *Actinobacillus pleuropneumoniae* and PRRSV infection, respectively [16,17]. Fibroblasts isolated from pigs overexpressing Mx1 were less vulnerable to influenza A virus and CSF virus infection [18], and cells from MxA TG pigs could suppress CSF virus replication [19].

Mammal defensins are the major group of cationic host defense peptides which are classified as three subfamilies, α-, β- and θ-defensins, according to their intramolecular disulfide bond pattern [20]. In terms of defensins in pigs, β-defensin is the only subgroup that has been found, with 27 functional β-defensins identified [21,22]. PBD-2 was first discovered by sequence similarity analysis with the well characterized porcine β-defensin 1 [23] and was proved to exist in different organs in pigs [16]. It has been shown that PBD-2 possesses antimicrobial abilities against a broad range of bacteria, both Gram-positive and Gram-negative [24]. The antibacterial mechanism of PBD-2 was elucidated in *Escherichia coli*, being disruption of the membrane integrity and affecting the DNA transcription and translation when PBD-2 entered into the cytoplasm [25]. Previous studies described that PBD-2 could hamper PRRSV replication in vitro [26] and suppress proliferation of pseudorabies virus both in vitro and in TG mice [27]. Besides, PBD-2 has been identified to alleviated inflammation by binding toll-like receptor 4 and inhibiting the subsequent NF-κB activation [28]. Additionally, PBD-2 has been used as a feed additive to improve growth performance, and to prevent post-weaning diarrhea in piglets [29]. Taken together, the *pbd-2* gene could be a promising candidate to generate disease-resistant TG animals.

Although pigs overexpressing PBD-2 have been produced by random gene integration in our previous study, a neomycin-resistance (*neoR*) gene has also been introduced into the pig genome [16]. Given that the CRISPR/Cas9 system is recognized as one of the most efficient tools for precise gene modifications in mammals [30,31], this study aimed to increase the expression level of PBD-2 in pigs by marker-free knock-in of *pbd-2* gene into the porcine *Rosa26* (*pRosa26*) locus, a safe harbor for ubiquitous expression of exogenous genes [32]. The resulting TG pigs would greatly improve resilience of pigs to infectious diseases, providing a potent genetic material for the breeding of disease-resistant pigs.

## 2. Materials and Methods 

### 2.1. Cells, Bacterial Strains and Animals

Porcine fetal fibroblasts (PFFs) and porcine ear fibroblasts (PEFs) were grown in Dulbecco’s Modified Eagle Medium (DMEM; Thermo Fisher Scientific, Waltham, MA, USA) supplemented with 20% fetal bovine serum (FBS; Thermo Fisher Scientific), while PK-15 cells (ATCC Number: CCL-33) were maintained in DMEM supplemented with 10% FBS. *Streptococcus suis* strain SC19 was cultured in tryptic soy broth (TSB; BD, Franklin Lakes, NJ, USA) with 5% newborn calf serum (NBCS; TIANHANG, Huzhou, China) and on tryptic soy agar (TSA; BD) with 5% NBCS. *A*. *pleuropneumoniae* strain 4074 was grown in TSB with 5% NBCS and 10 μg/mL of nicotinamide adenine dinucleotide (NAD; Sigma-Aldrich, St. Louis, MO, USA) and on TSA with 5% NBCS and NAD. Experiments involving pigs were performed in accordance with the Hubei Regulations for the Administration of Affairs Concerning Experimental Animals and were approved by the Scientific Ethical Committee for Experimental Animals of Huazhong Agricultural University, Wuhan, China (HZAUSW-2019-010).

### 2.2. Plasmids

**Figure 1:**
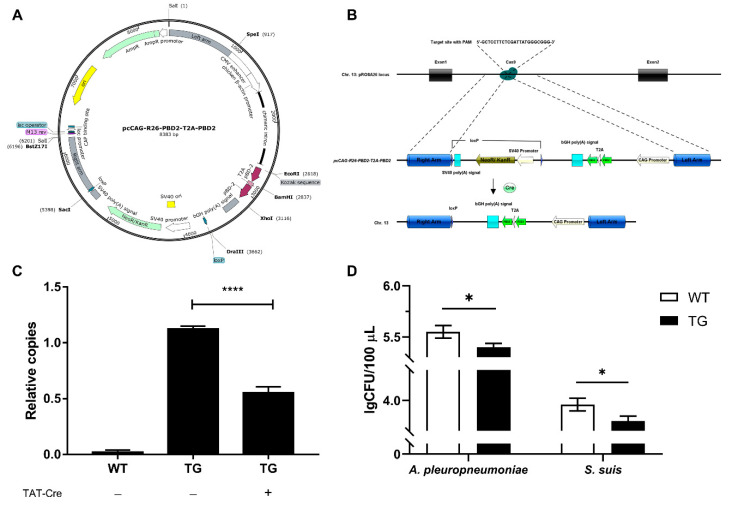
Site-specific *pbd-2* knock-in at the porcine *Rosa26* (*pRosa26*) locus. (**A**) Map of the donor plasmid *pcCAG-R26-PBD2-T2A-PBD2* for the site-specific *pbd-2* knock-in. (**B**) Scheme for marker-free targeted *pbd-2* integration in porcine fetal fibroblasts (PFFs) via CRISPR/Cas9-mediated homology-directed repair. The floxed selectable marker gene (SMG) was removed after treatment of Cre recombinase. (**C**) Relative copy number of the SMG *neoR* in wild-type (WT) PFFs, transgenic (TG) PFFs and Cre-recombinase-treated TG PFFs. (**D**) The bactericidal activities of cell culture supernatants of WT PFFs and TG PFFs on *Actinobacillus pleuropneumoniae* and *Streptococcus suis* quantified by bacterial counting. Data are presented as mean ± SD and are plotted from three independent experiments. * *p* < 0.05, **** *p* < 0.0001, unpaired one tailed Student′ s *t*-test.

An efficient Kozak sequence (5′-GCCACC-3′) was added upstream of the ATG start codon of the *pbd-2* gene in *pcCAG-PBD2* vector constructed in our previous study [16], with the resulting vector named as *pcCAG-nPBD2* (Figure S1A). To evaluate whether the 2A peptide system could be efficient in linking two copies of *pbd-2* gene, *pcCAG-PBD2-T2A-PBD2* was produced, containing two copies of *pbd-2* gene linked by a T2A peptide sequence (Figure S1B). A 5′ homology arm (HA) of approximately 0.9 kb and a 3′ HA of approximately 0.76 kb was cloned into the *Spe*I and *Bst*Z17I site of *pcCAG-PBD2-T2A-PBD2*, respectively. Subsequently, two loxP sites with the same orientation were inserted, flanking the selectable marker gene (SMG) *neoR*. The resulting vector was designated as *pcCAG-R26-PBD2-T2A-PBD2* (Figure 1A). The *pX330-pRosa26* vector (Figure S2) provided by Professor Bo Zuo (Huazhong Agricultural University) was used to express Cas9 and sgRNA targeting the *pRosa26* locus (5′-GCTCCTTCTCGATTATGGGC-3′).

### 2.3. Transfection of PK-15 Cells

At 80% confluency, PK-15 cells were respectively transfected with *pcCAG-nPBD2* and *pcCAG-PBD2-T2A-PBD2* using the Lipofectamine 3000 (Thermo Fisher Scientific) according to the manufacturer’s instruction. After 24 h the medium was replaced with fresh DMEM containing 1% FBS. The cell culture supernatant was collected 48 h later for the subsequent bactericidal assay against *A. pleuoropneumoniae*. 

### 2.4. Bactericidal Assay

A 10 μL aliquot of *S. suis* (5000 CFU) or *A. pleuropneumoniae* (5000 CFU) was incubated with 90 μL of cell culture supernatants at 37 °C for 1 h, respectively. The mixture was then serially diluted (1:100 and 1:1000 dilution) in DPBS (Thermo Fisher Scientific) and spread onto TBA plates. The plates were left at 37 °C overnight and colonies were counted and analyzed. Bacteria incubated with DPBS was used as a blank control, while those incubated with 60 μg/mL of the synthetic mature PBD-2 (DHYICAKKGGTCNFSPCPLFNRIEGTCYSGKAKCCIR) (ChinaPeptides, Shanghai, China) diluted in DPBS served as a positive control. 

### 2.5. Isolation of Genomic DNA

Cells or pig ear tissues were incubated in 500 μL of lysis buffer (10 mM Tris, pH 8.0; 10 mM NaCl; 10 mM EDTA, pH 8.0; 10% SDS; 400 μg/mL Proteinase K) at 56 °C for 8 h. After 200 μL of saturated NaCl was added, tubes were gently shaken for 5 min before centrifugation 13,000× *g* for 10 min. The supernatant was then transferred to a new tube and equal volume of isopropanol (chilled at −20 °C) was added. The mixture was incubated at −20 °C for another 2 h and was subjected to centrifugation 13,000× *g* for 10 min. The supernatant was discarded and the precipitate was washed with 70% ethanol. The tube was then centrifugated 13,000× *g* for another 10 min and the supernatant was removed. After air dry at room temperature (RT) for 10 min, the resulting DNA was resuspended in pre-heated ddH_2_O and the concentration was measured using a spectrophotometer (Thermo Fisher Scientific).

### 2.6. Electroporation and Selection of PFFs

PFFs were cultured in a 100-mm dish for two days to reach 80% confluency. After washing with DPBS twice, PFFs were detached from the dish using 0.25% Trypsin-EDTA (Thermo Fisher Scientific) and were centrifugated at 1000× *g* for 5 min. The cell pellet was suspended in 800 μL of Gene Pulser Electroporation Buffer (Bio-Rad, Hercules, CA, USA) and 32 μg of *pcCAG-R26-PBD2-T2A-PBD2* and *pX330-pRosa26* each were added and gently mixed. Cell suspension was added into a 0.2 cm-gap Gene Pulser Electroporation Cuvettes (200 μL) for the subsequent electroporation using the Gene Pulser Xcell system (Bio-Rad) and was left recovery for 24 h. Following selection in 400 μg/mL of G418 for two weeks, single colonies were picked and transferred onto a 48-well plate. As the cells reached confluency, half of them were subjected to genomic DNA isolation which was later used as the PCR template for genotyping. The site-specific knock-in events were identified by PCR using three primer pairs (Table S1) and the positive cell clone was then chosen for the removal of a SMG.

### 2.7. Removal of Selectable Marker

When cells reached about 60% confluency, cells were treated with 3 μM TAT-Cre recombinase (Merck Millipore, Burlington, MA, USA) in DMEM without the presence of antibiotics and FBS. After 3 h of incubation at 37 °C, the medium was removed and the cells were washed twice with PBS. The treated cells were grown in fresh medium and 50% of them were subjected to somatic cell nuclear transfer (SCNT). The remaining cells were used to determine the recombination efficiency by real-time quantitative PCR (RT-qPCR). Briefly, genomic DNA from wild-type (WT) cells, treated and untreated TG cells was extracted and subjected to RT-qPCR using SYBR^®^ Green Realtime PCR Master Mix (TOYOBO, Osaka, Japan). The relative copy number of the SMG *neoR* was analyzed using the 2^−ΔΔCt^ method [33], with *β-actin* as the reference gene. Specific primers for *neoR* are NeoR-F (5′-GCCCCATGGCTGACTAATTTTTTTT-3′) and NeoR-R (5′-CGATTGTCTGTTGTGCCCAGTC-3′), while primers for *β-actin* are ACTB-F (5′-GCCTCTCGTCTTGCTTGTTTTAAA-3′) and ACTB-R (5′-AGCAAGTGAGGGCGTATCCAG-3′). Recombination efficiency = (mean value of treated TG group − mean value of WT group)/(mean value of untreated TG group − mean value of WT group) × 100%. Meanwhile, the cell culture supernatants of WT cells and treated TG cells were collected to measure the bactericidal activity against *A. pleuoropneumoniae* and *S. suis* as described above.

### 2.8. Generation and Genotype Analysis of Cloned Piglets

After removal of the SMG, positive PFFs were subjected to the procedure of SCNT by ViaGen Animal Breeding Resources Development Company (Wuhan, China) in accordance with the protocol described elsewhere [34]. There were eight surrogate sows used and each was transferred with 250 reconstructed embryos.

The resulting piglets were identified by PCR. Genomic DNA was obtained from pig ear tissues of the newborn piglets as described above. Four pairs of primers (Table 1) were used to identify marker-free TG pigs and the PCR products were then sequenced for further confirmation. The Southern blot analysis was performed to confirm a site-specific transgene integration into the pig genome. Briefly, genomic DNA of high-quality was isolated from the PEFs using the QIAamp DNA Mini Kit (QIAGEN, Hilden, Germany) and digested with *Xho*I. The digested DNA was electrophoretically separated on an agarose gel and then transferred onto a nylon membrane. The probe labeled with digoxigenin was hybridized to a single DNA fragment on the membrane, indicating a site-specific insertion of duplicate *pbd-2* genes.

### 2.9. Immunofluorescent and Immunohistochemical Analysis for PBD-2 

PEFs were isolated from the ear tissue of cloned pigs and were subjected to the subsequent immunofluorescent analysis. Same amounts of WT and TG PEFs (10,000 cells each) were firstly grown on coverslips for 24 h before being rinsed in PBS, then cells were incubated in chilled acetone (−20 °C) for 10 min. The coverslips were washed three times with ice-cold PBS before being blocked with 1% BSA, 22.52 mg/mL glycine in PBST (PBS+ 0.1% Tween 20) for 30 min. The PEFs were then incubated in self-made PBD-2 mouse monoclonal antibody [16] diluted in 1% BSA in PBST at RT for 1 h. Subsequently, cells were washed five times with PBST for 5 min each and were then incubated with FITC-labeled Goat Anti-Mouse IgG (H+L) (Abclonal, Wuhan, China) diluted at 1:100 in 1% BSA in PBST for 1 h without exposure to light. After that, cells were washed five times with PBST for 5 min each in the dark and then were stained with 4′,6-diamidino-2-phenylindole (Beyotime, Shanghai, China). The results were observed using the EVOS FL Auto Imaging System (Thermo Fisher Scientific).

Besides, a TG founder pig and a WT littermate were both sacrificed to harvest their heart, liver, spleen, lung, kidney, and brain tissues. Tissues were fixed in PBS-buffered 4% formaldehyde followed by paraffin embedding and sectioning. The sections were then subjected to immunohistochemical analysis to detect PBD-2 in different organs. Mouse anti-2A peptide monoclonal antibodies (Novus Biologicals, Littleton, CO, USA) and HRP-conjugated goat anti-mouse IgG were used as primary and secondary antibodies, respectively. PBD-2 was detected after diaminobenzidine staining as a brown coloration.

### 2.10. Transcriptional Analysis of pbd-2 Gene in TG Pigs Using RT-qPCR

Total RNA from heart, liver, spleen, lung, kidney and brain of the TG and WT pigs was extracted using RNAiso Plus (TAKARA, Dalian, China). The obtained RNA of 500 ng from different organs was then reverse-transcribed into cDNA for the subsequent RT-qPCR to measure the relative mRNA levels of *pbd-2*, with *GAPDH* as the reference gene. Specific primers for *pbd-2* are tPBD2-F (5′-AGAGGGCAGAGGAAGTCTGCTAA-3′) and tPBD2-R (5′-TTTAAACGGGCCCTCTAGACTCGA-3′), while primers for *GAPDH* are GAPDH-F (5′-ACCCAGAAGACTGTGGATGGC-3′) and GAPDH-R (5′-AGCCAGAGGCAAAGTGATAGATA-3′).

### 2.11. Off-Target Analysis

Potential off-target sites in pig genome were predicted using an online tool Cas-OFFinder [35]. The maximal mismatch number was set as three, with PAM type being 5′-NGG-3′. After that, fragments containing predicted off-target sites were PCR amplified using primer pairs in Table S2 and subjected to Sanger sequencing for sequence alignment. 

### 2.12. Statistical Analysis

Data were analyzed with GraphPad Prism 8 (GraphPad Software, La Jolla, CA, USA) using unpaired one-tailed Student’s *t*-test and shown as mean ± SD. * *p* < 0.05, ** *p* < 0.01, *** *p* < 0.001, **** *p* < 0.0001.

## 3. Results

### 3.1. Enhanced Bactericidal Activity by T2A-Linked Dual pbd-2 Expression

To achieve simultaneous expression of two copies of *pbd-2* gene, a T2A self-cleaving peptide sequence was put between two *pbd-2* genes in *pcCAG- PBD2-T2A-PBD2* (Figure S1B). Cell culture supernatants from PK-15 cells transfected with *pcCAG-PBD2-T2A-PBD2* and the control vector *pcCAG-nPBD2* carrying a single copy of *pbd-2* (Figure S1A) were collected and incubated with the same number of *A. pleuropneumoniae* for 1 h, respectively. As shown in Figure S1C, the number of surviving bacteria after incubating with cell culture supernatant of cells transfected with *pcCAG-PBD2-T2A-PBD2* was significantly less than that with *pcCAG-nPBD2*, indicating that T2A-mediated dual *pbd-2* expression could significantly increase the bactericidal activity of cell culture supernatants.

### 3.2. CRISPR/Cas9 and Cre/loxP-Mediated Marker-Free Site-Specific Insertion of pbd-2 in PFFs

The *pcCAG-R26-PBD2-T2A-PBD2* (Figure 1A) along with *pX330-pRosa26* (Figure S2) were co-electroporated into PFFs to achieve the integration of dual *pbd-2* genes, as well as a SMG *neoR*, into the *pRosa26* site. After G418 screening, positive cell colonies was identified by PCR (Figure S3) and the treatment of TAT-Cre recombinase significantly decreased the copy number of *neoR* in TG cells (Figure 1B,C), reaching a recombination efficiency of 48.3% as calculated in accordance with the method described above. In the meantime, the cell culture supernatant of treated TG cells displayed significant increase in the bactericidal activity against both *S. suis* and *A. pleuropneumoniae* (Figure 1D). These indicated that 48.3% of the TG cells were marker-free and exerted enhanced antibacterial activity, suggesting an increase in PBD-2 expression in the treated TG cells, which could be used for the subsequent SCNT.

### 3.3. Genotyping of Cloned Piglets

**Figure 2:**
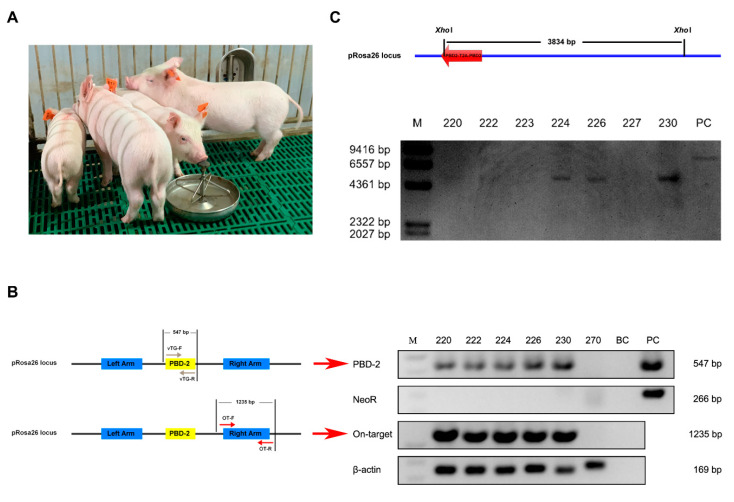
Genotyping of cloned piglets. (**A**) Physical appearance of cloned piglets. (**B**) PCR analysis to identify marker-free site-specific *pbd-2* knock-in pigs. PBD-2: Amplification for the dual *pbd-2* gene using primers vTG-F and vTG-R; NeoR: Amplification for the SMG *neoR*; Off-target: Amplification for the fragment which represents on-target insertion of the transgene using primers OT-F and OT-R; β-actin: Amplification for *β-actin*; Lane 220–227: Numbers for pigs; BC: Blank control; PC: Positive control. (**C**) Southern blot analysis for the identification of TG pigs. Genomic DNA from porcine ear fibroblasts (PEFs) was extracted and digested with *Xho*I, followed by Southern blot analysis using a digoxigenin-labeled *pbd-2*-specific probe. M: Molecular mass marker; Lane 220–230: Numbers for pigs; PC: Positive control.

After transferring 250 reconstructed embryos into the uterus of each recipient, there were three surrogate sows successfully giving birth to seven piglets in total. All the cloned piglets had normal physical appearance when compared with WT pigs (Figure 2A). Regarding identification of TG pigs based on PCR results, the observed bands for the PBD-2 and On-target fragments represented a site-specific *pbd-2* knock-in at the *pRosa26* locus, while no band for the NeoR fragment indicated that the SMG *neoR* was not introduced into the pig genome. As shown in Figure 2B, there were five pigs harboring a site-specific *pbd-2* at the *pRosa26* locus, with no SMG detected. The on-target integration of *pbd-2* in TG pigs was validated by Sanger sequencing analysis on the resulting PBD-2 and On-target fragments (Figure S4). The Southern blot analysis was carried out to further confirm a targeted *pbd-2* insertion into the pig genome. Consistent with the PCR results, there were intended bands for pigs numbered as 220, 222, 224, 226 and 230 (Figure 2C), indicating a site-specific integration of duplicate *pbd-2* genes in these pigs. Together, we obtained five marker-free pigs with the *pbd-2* gene site-specifically incorporated at the *pRosa26* locus.

### 3.4. Off-Target Analysis

**Figure 3:**
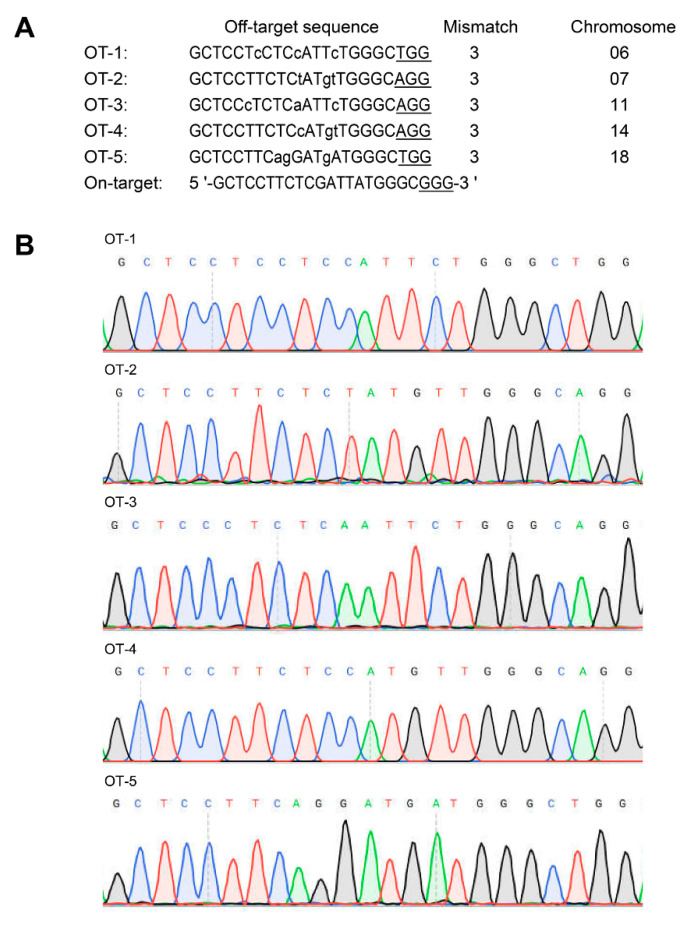
Off-target analysis. (**A**) Predicted off-target sites. Lower case letters represent mismatched bases, while underlined letters represent the 3′ PAM of the target sequence. (**B**) Sanger sequencing results of the PCR products of potential off-target sites.

The potential off-target sites were predicted according to the target sequence (5′-GCTCCTTCTCGATTATGGGCGGG-3′) using Cas-OFFinder. The results showed that there were five potential off-target sites on chromosome 06, 07, 11, 14, 18, respectively (Figure 3A). The fragments containing all these potential off-targets loci were PCR amplified and sequenced. The Sanger sequencing results revealed that no insertions/deletions and site mutations were found within all these potential off-target sites (Figure 3B). These findings suggested no potential off-target effects were observed in TG pigs.

### 3.5. Characterization of PBD-2 Transcription and Translation

**Figure 4:**
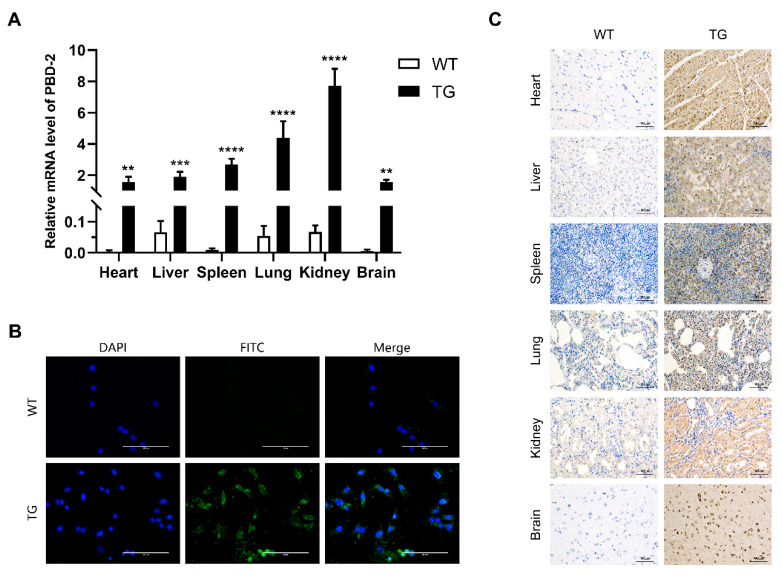
Transcriptional and translational analysis of *pbd-2* in cloned pigs. (**A**) The mRNA expression level of *pbd-2* in different organs. Total RNA in different organs of TG and WT pigs was extracted and then subjected to reverse transcription, followed by RT-qPCR to determine the mRNA level of *pbd-2*, *GAPDH* was used as an internal reference gene. Data are presented as mean ± SD and are plotted from three independent experiments. ** *p* < 0.01, *** *p* < 0.001, **** *p* < 0.0001, *n* = 3, unpaired one tailed Student′ s *t*-test. (**B**) Immunofluorescent analysis of the expression of porcine β-defensin 2 (PBD-2) using a 40× objective and a 100 ms exposure. The expression of PBD-2 in PEFs from TG pigs was detected by immunofluorescent analysis using mouse monoclonal anti-PBD2 antibody followed by FITC-labeled goat anti-mouse IgG. Cell nuclei were stained with 4′,6-diamidino-2-phenylindole, the PEFs from WT pigs served as a negative control. Scale bar = 100 μm. (**C**) Immunohistochemical analysis of the expression of PBD-2 at a magnification of 200×, with an exposure time of 50 ms. Expression of PBD-2 in heart, liver, spleen, lung, kidney and brain tissues of WT and TG pigs was determined by immunohistochemistry using mouse monoclonal 2A peptide antibody. Brown color represents the detected 2A peptide, which is co-expressed with PBD-2. Scale bars = 100 μm.

The transcriptional level of *pbd-2* expression in different organs of TG pigs was quantified by RT-qPCR, with *GAPDH* serving as the reference gene. As shown in Figure 4A, the mRNA levels of *pbd-2* in heart, liver, spleen, lung, kidney and brain tissues of TG pigs were significantly higher than that of their WT littermates. Besides, the kidney showed the highest mRNA level of *pbd-2*, while the transcriptional level of *pbd-2* of lung and spleen ranked the second and the third, respectively.

To further determine whether the CRISPR/Cas9-mediated knock-in of *pbd-2* could increase the expression of PBD-2, the immunofluorescent analysis was conducted to detect PBD-2 in PEFs isolated from WT and TG pigs, using the self-made mouse anti-PBD-2 monoclonal antibody. The results showed high expression of PBD-2 in TG PEFs, with strong green fluorescence. In contrast, little green signals could be seen in WT PEFs, indicating low expression of PBD-2 in WT pigs (Figure 4B). The immunohistochemical analysis was further performed to detect PBD-2 in different organs of both WT and TG pigs. To exclude the influence of endogenous expression of PBD-2, an anti-2A peptide antibody was used as the primary antibody for that 2A peptide and PBD-2 were co-translated. As was shown in Figure 4C, PBD-2 in different organs of TG pigs was highly expressed, appearing as brown signals, while no obvious brown coloration was observed in tissues of WT pigs, which was in good agreement with those results identified by immunofluorescence and RT-qPCR.

### 3.6. Verification of the Antibacterial Ability of Isolated TG PEFs

**Figure 5:**
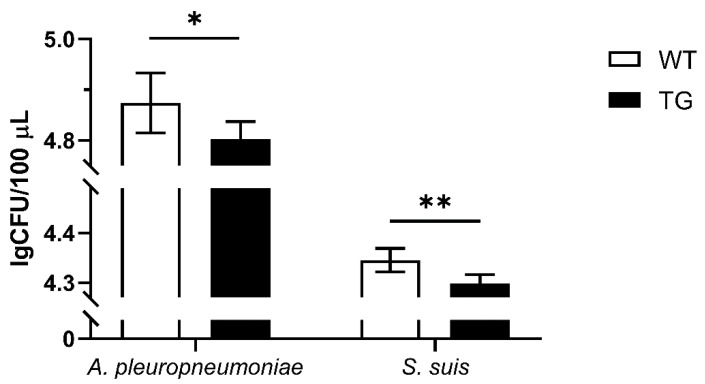
Bactericidal activity of cell culture supernatant. The cell culture supernatant of PEFs from TG pigs was incubated with *A. pleuropneumoniae* and *S. suis* for 1 h and the surviving bacteria were then plated on agar for counting. Cell culture supernatant of PEFs from WT pigs was used as a negative control. Data are presented as mean ± SD and are plotted from three independent experiments. * *p* < 0.05, ** *p* < 0.01, unpaired one tailed Student′ s *t*-test.

To investigate whether overexpression of PBD-2 could endow hosts with enhanced resistance to bacterial infections, PEFs were isolated from TG and WT pigs. Then cell culture supernatants of WT and TG PEFs were harvested to incubate with *A. pleuoropneumoniae* and *S. suis*, and the bactericidal activity of the supernatants was analyzed by bacterial counting. The surviving bacteria of the TG group were significantly less than that of the WT group (Figure 5), suggesting that the site-specific knock-in of *pbd-2* successfully conferred an improved resistance against bacterial infections on TG pigs. 

## 4. Discussion

As a major group of cationic peptides widely existing in animals, defensins have been proven to possess broad antimicrobial activities against different pathogens including bacteria, fungi and viruses. Our previous in vivo studies showed that pigs overexpressing PBD-2 displayed increased resistance to *A. pleuoropneumoniae* infection [16], while mice expressing PBD-2 became more resistant to pseudorabies virus and *Salmonella* Typhimurium infections [27,28]. Given that the previous *pbd-2* TG pigs were produced using random gene insertion, which also introduced a SMG, these would raise public concern on the biosafety of TG animals and hamper the commercialization of TG animals derived products. Therefore, this study achieved site-specific integration of *pbd-2* into the *pRosa26* locus through CRISPR/Cas9-mediated homology-directed repair and eliminated the SMG *neoR* using a cell-penetrating TAT-Cre recombinase. Five marker-free *pbd-2* knock-in pigs were generated via SCNT, making it to the overexpression of PBD-2 in different organs of pigs with no off-target effects detected. Additionally, the cell culture supernatant of PEFs of TG pigs exhibited greater antibacterial activities against both Gram-negative (*A. pleuoropneumoniae*) and Gram-positive bacterium (*S. suis*).

The copy number of genes is positively correlated with corresponding protein levels [36]. Fellermann et al. claimed that people carried low copy number of human β-defensin 2 gene were more likely to develop Crohn’s disease [37], while a high copy number of β-defensin genes was considered the main reason why East Asians were more resistant to influenza infection [38]. As the 2A self-cleaving peptide has been widely used to generate multi-transgenic pigs [39], we applied T2A peptides, a 2A self-cleaving peptide found in Thosea asigna virus 2A [40], in expression of two copies of the *pbd-2* gene in this study. Similar with other defensins, PBD-2 is firstly produced in the form of pro-peptide and then processed into active form when the N-terminal signal peptide is cleaved, followed by being transported out of cells [16]. Though de Felipe and Ryan claimed that proteins downstream of the 2A peptide could also occur on cell membrane or be secreted despite lack of a signal sequence when a protein upstream of the 2A peptide contained an N-terminal signal sequence [41], while Yan et al. argued that genes following the 2A peptide still demanded a signal sequence for successful secretion expression [42]. Thus, the signal sequence in the second *pbd-2* gene was kept and the bactericidal assay showed that this design could efficiently improve the expression of PBD-2 without hampering its biological activity. 

The random integration of exogenous genes has been found to lead to multiple undesired outcomes including gene silencing, unpredictable expression and activation of oncogene expression [43]. In addition to the *pRosa26* locus described above, porcine *H11* and *GAPDH* loci have been well characterized as safe harbors for targeted transgene integrations [44,45]. When introducing a foreign gene into the *Rosa26* locus, higher expression could be achieved by positioning the transgene promoter in an opposite direction to the *Rosa26* promoter [46]. Therefore, this study set *pbd-2* gene in a reverse orientation to the *Rosa26* promoter and achieved overexpression of PBD-2.

The unwanted effects of introducing a SMG *neoR* have been well investigated. Wang et al. argued that the *neoR* gene could inhibit the growth of *Lactobacillus* and *Escherichia-Shigella-Hafnia* in guts of TG pigs [47]. Meanwhile it was worth noting that the expression levels of metabolic genes varied between cells expressing exogenous *neoR* gene and control cells [48]. Also, the integration of *neoR* gene in target loci has been shown to exert a long-distance effect on the expression of downstream or upstream genes [49]. Another study revealed that removing the selectable marker increased the expression level of transgene when promoters of the *Rosa26* and the transgene occurred in opposite orientations [46]. Bi et al. successfully excised the SMG in cells using Cre mRNA [4], while using cell-penetrating TAT-Cre recombinase to delete a SMG could reach a recombination efficiency of up to 55% in pig primary cells [50]. Alike, the use of commercialized TAT-Cre recombinase in this study achieved successful excision of *neoR* in 48.3% PFFs.

It has been quite controversial that whether CRISPR/Cas9-mediated gene-editing can cause genome-wide off-target mutations, while Zuo et al. created a large scale of screening system called GOTI and used to prove that classic CRISPR/Cas9 tool did not induce obvious off-target effects [51]. This study predicted five putative off-target loci using an online tool and the Sanger sequencing of the five sites in TG pigs revealed that no mutations were found among these sites. However, in-depth whole-genome sequencing of the TG pigs is still needed to exclude any off-target mutagenesis in the future.

Chen et al. found that, when compared with crossbred pigs, higher expression of β-defensins in Meishan pigs was one of the main reasons that Meishan pigs exhibited effective disease-resistance traits [52]. The RT-qPCR analysis combined with immunohistochemical and immunofluorescence assays had confirmed that PBD-2 was overexpressed in different organs of the marker-free *pbd-2* knock-in pigs (Figure 4), while the bactericidal assay indicated that PEFs of TG pigs were more resistant to bacterial infections (Figure 5). In addition to the described functions of PBD-2 above, the in vitro studies have shown that PBD-2 could inhibit proliferations of PRRSV, *Staphylococcus aureus* and other pathogens [24,26]. Besides, PBD-2 could be used for growth-promotion among piglets [29,53]. Altogether these suggest excellent prospect for the application of marker-free site-specific *pbd-2* knock-in pigs produced in this study. 

In summary, this study generated marker-free site-specific *pbd-2* knock-in pigs, achieving overexpression of PBD-2 in pigs, which provides a novel genetic material for the breeding of disease-resistant pigs. Given that PBD-2 displays multi-functions including antimicrobial and immunomodulatory activities, these pigs are supposed to be reliable animal models to study biofunctions of PBD-2 as well.

## Figures and Tables

**Table 1:** Information of primer pairs for genotype analysis of cloned pigs.

Fragment	Primer	Sequence (5′-3′)	Length (bp)
PBD-2	vTG-F	GCTGGTTGTTGTGCTGTCTCATCA	547
	vTG-R	CCCTCTAGACTCGAGTCAGGGTCAGC	
On-target	OT-F	CTTCCTTTCTCGCCACGTTC	1235
	OT-R	TCGGTAAATAGCAATCAACTCAG	
NeoR	NeoR-F	GCCCCATGGCTGACTAATTTTTTTT	266
	NeoR-R	CGATTGTCTGTTGTGCCCAGTC	
β-actin	Control-F	GCCTCTCGTCTTGCTTGTTTTAAA	169
	Control-R	AGCAAGTGAGGGCGTATCCAG	

## Supplementary Materials

The following are available online at https://www.mdpi.com/2073-4425/11/8/951/s1, Figure S1: Analysis for the T2A-mediated dual *pbd-2* expression, Figure S2: Map of *pX330-pRosa26*, Figure S3: PCR analysis for the identification of site-specific *pbd-2* knock-in PFFs, Figure S4: Sanger sequencing analyses to confirm on-target integration of *pbd-2* in cloned pigs, Table S1: Information of primer pairs for identification of transgenic PFFs, Table S2: Information of primer pairs for amplification of predicted off-target loci.
