# Supplementary material for: Generation of Marker-Free pbd-2 Knock-in Pigs Using the CRISPR/Cas9 and Cre/loxP Systems
Source: Genes (Basel). 2020 Aug 18;11(8):951. doi: 10.3390/genes11080951 (PMC7465224; doi:10.3390/genes11080951)
Supplement: Supplementary file 1 [file genes-11-00951-s001.pdf]

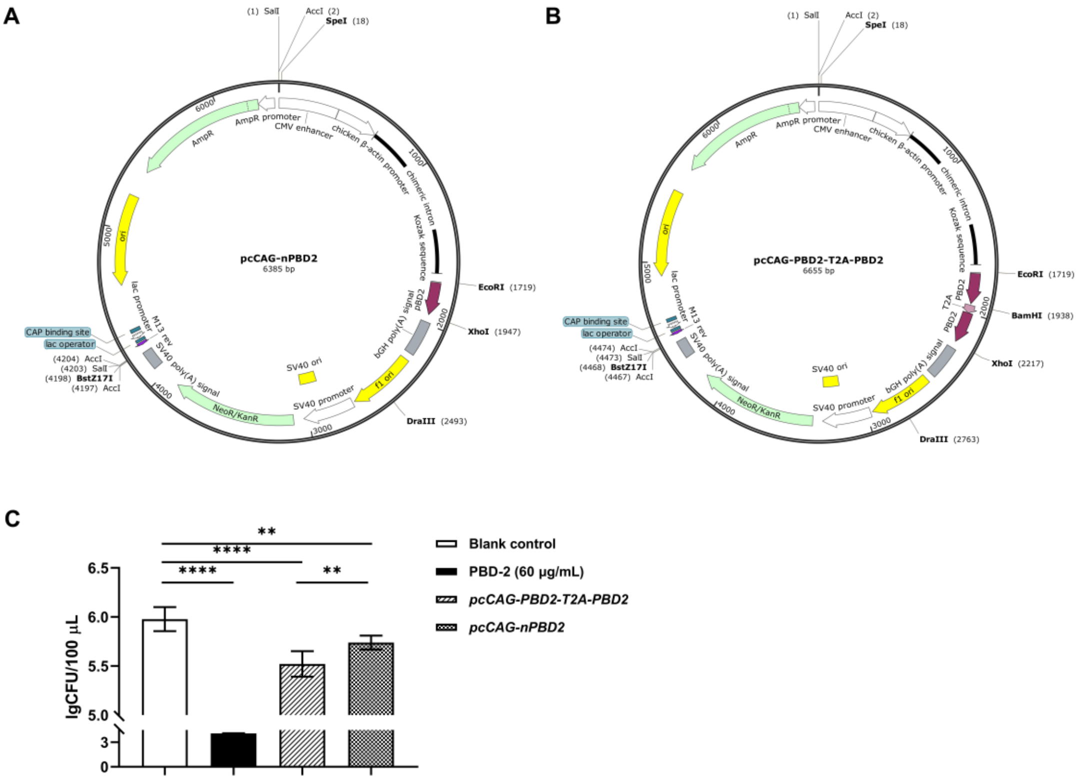


**Figure S1.** Analysis for the T2A-mediated dual *pbd-2* expression. (**A**) Map of *pcCAG-nPBD2*. (**B**) Map of *pcCAG -PBD2-T2A-PBD2*. (**C**) PK-15 cells were transfected with *pcCAG-nPBD2* and *pcCAG-PBD2-T2A-PBD2*, respectively. The cell culture supernatant was collected 48 h after transfection and then was incubated with *Actinobacillus pleuropneumoniae* for 1 h, the surviving bacteria were then plated on agar for counting. Cell culture supernatant of PK-15 was used as a blank control and porcine β-defensin 2 (PBD-2) of 60 μg/mL served as a positive control. Data are presented as mean ± SD and are plotted from three independent experiments. $^{**}P < 0.01$, $^{****}P < 0.0001$, unpaired one tailed Student' s *t*-test.

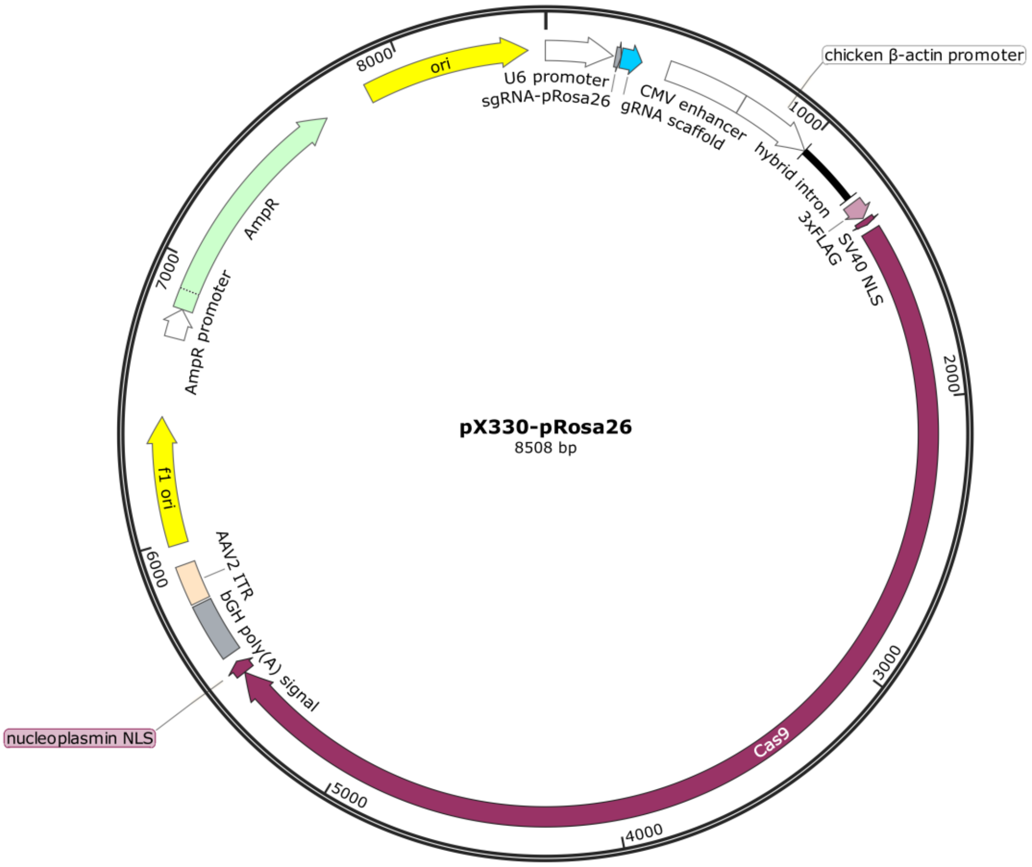


**Figure S2.** Map of *pX330-pRosa26*. A plasmid expressing Cas9 nuclease and sgRNA targeting the *pRosa26* locus (5′-GCTCCTTCTCGATTATGGGC-3′).

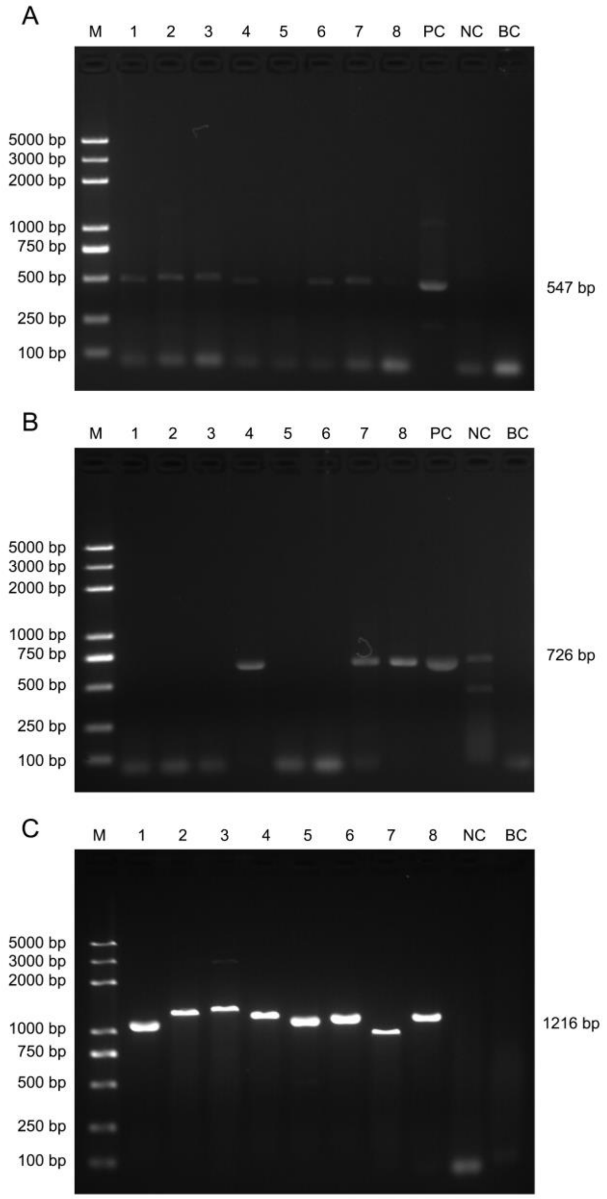


**Figure S3.** PCR analysis for the identification of site-specific *pbd-2* knock-in porcine fetal fibroblasts (PFFs). (**A**) Amplification for the dual *pbd-2* gene; (**B**) Amplification for the selectable marker gene *neoR*; (**C**) Amplification for the fragment which represents on-target insertion of the transgene. M: Molecular mass marker; Lane 1-8: Numbers for different cell colonies; PC: Positive control; NC: Negative control; BC: Blank control.

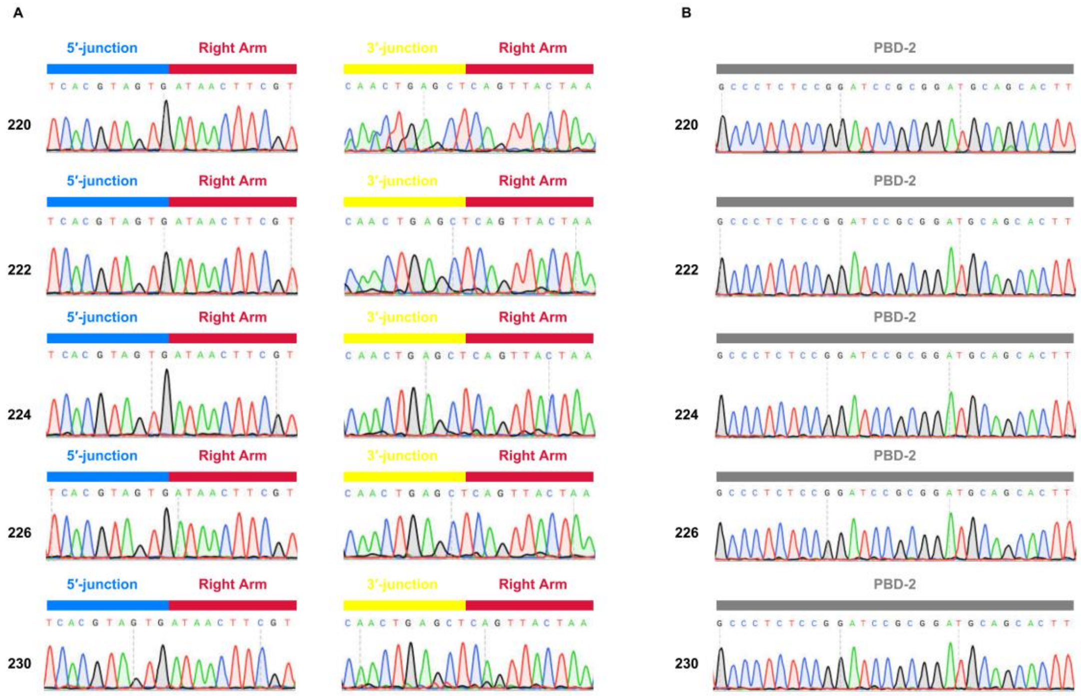


**Figure S4.** Sanger sequencing analyses to confirm on-target integration of *pbd-2* in cloned pigs. Numbers represent different transgenic pigs. (**A**) Sequencing results of the On-target fragment amplified with primers OT-F and OT-R. Red line indicates the sequence in the right homology arm; blue line indicates the sequence upstream of the right homology arm; yellow line indicates the sequence downstream of the right homology arm. (**B**) Sequencing results of the PBD-2 fragment amplified with primers vTG-F and vTG-R. Gray line indicates the sequence in the *pbd-2* gene.

**Table S1.** Information of primer pairs for identification of transgenic PFFs.

| **Fragment** | **Primer** | **Sequence (5′-3′)** | **Length (bp)** |
|---|---|---|---|
| PBD-2 | vTG-F | GCTGGTTGTTGTGCTGTCTCATCA | 547 |
| | vTG-R | CCCTCTAGACTCGAGTCAGGGTCAGC | |
| Marker | marker-F | GATGGATTGCACGCAGGTTCTCC | 726 |
| | marker-R | GATACCGTAAAGCACGAGGAAGCG | |
| Locus | f-locus | CCTTCTATCGCCTTCTTGACGAGTT | 1216 |
| | r-locus | AACTGTCCCAGCAACACCTAAGATT | |

**Table S2.** Information of primer pairs for amplification of predicted off-target loci.

| Fragment | Primer | Sequence (5′-3′) | Length (bp) |
|---|---|---|---|
| OT-1 | OT1-F | ATAAAACAATGGGGGAATGAGAC | 429 |
| | OT1-R | AGCCAGAGGCAAAGTGATAGATA | |
| OT-2 | OT2-F | CACAGAAGGAATTCCCTGAGAGA | 389 |
| | OT2-R | CATGCTTCTCTGCACACACTA | |
| OT-3 | OT3-F | TCGGTGTCCTTGCCAAGTCG | 417 |
| | OT3-R | CACCTTCCTGGCATTAATCCCT | |
| OT-4 | OT4-F | AGTTAATGATTCCTAGGACATGCTT | 367 |
| | OT4-R | TCAAAGTGGCGGGTAAGGGAA | |
| OT-5 | OT5-F | GGGTCTGTGTTGTTGCATGAGAA | 350 |
| | OT5-R | CACAGTGGGTGAGGTAAGAAATT | |
